# Phytate Effects on Incomplete Distal Renal Tubular Acidosis

**DOI:** 10.3390/jcm13175059

**Published:** 2024-08-26

**Authors:** Jordi Guimerà, Ana Martínez, José Luis Bauzá Quetglas, Pilar Sanchis, Antonia Costa-Bauzá, Enrique Pieras, Felix Grases

**Affiliations:** 1 Urology Department, Health Research Institute of the Balearic Islands (IdISBa), Son Espases University Hospital, 07120 Palma de Mallorca, Spain; jorge.guimera@ssib.es (J.G.); anai_martinez@ssib.es (A.M.); jose.bauza@ssib.es (J.L.B.Q.); enriquec.pieras@ssib.es (E.P.); 2 Laboratory of Renal Lithiasis Research, University Institute of Health Sciences Research (IUNICS), University of Balearic Islands, 07122 Palma de Mallorca, Spain; pilar.sanchis@uib.es (P.S.); fgrases@uib.es (F.G.)

**Keywords:** phytate, distal renal tubular acidosis, hypercalciuria, urolithiasis

## Abstract

**Background:** Adults who have incomplete distal renal tubular acidosis (dRTA) may present with recurrent urolithiasis due to metabolic acidosis, leading to bone resorption, which in turn causes hypercalciuria and urine alkalinization (pH > 6.0). Oral potassium citrate is the most commonly used treatment for dRTA, but some patients cannot tolerate this treatment. The objective of this single-arm study was to evaluate the effect of phytate, an inhibitor of bone resorption, on calciuria of patients with incomplete dRTA. **Methods:** The calciuria levels of 16 patients who had incomplete dRTA with urolithiasis and could not tolerate potassium citrate treatment were recorded before (baseline) and after 6 months of treatment with oral calcium magnesium phytate (380 mg every 12 h). There were no dietary modifications or other treatments. **Results:** The baseline calciuria was 317 ± 81 mg/24 h and the level after 6 months was 221 ± 38 mg/24 h (*p* < 0.005). **Conclusions:** Our results suggest that calcium magnesium phytate should be considered as an alternative or adjunctive treatment for hypercalciuria in patients with incomplete dRTA.

## 1. Introduction

Urolithiasis has a high prevalence, affecting about 15% of people worldwide [1]. Distal renal tubular acidosis (dRTA), also known as renal tubular acidosis type 1, is a hereditary, idiopathic, or acquired disease [2] that is characterized by renal tubule dysfunction and decreased secretion of acid into the urine, leading to a condition of metabolic acidosis and urinary alkalosis (pH > 6). There are two forms of dRTA: complete dRTA is the most severe form, mainly occurs during childhood, has severe symptoms, and is associated with a short life expectancy; incomplete dRTA is a much milder disease that is usually diagnosed in adults with recurrent urolithiasis [3]. During metabolic acidosis in these patients, bone tissues undergo increased resorption, and this leads to the release of calcium into the blood, which has a buffering effect. The increased calcium in the blood is released into the urine, causing hypercalciuria, and an increase in the urinary pH to above 6. The high pH and high level of calcium in the urine greatly increase the risk of urolithiasis [3,4]. Calcium phosphate stones and calcium phosphate/calcium oxalate dihydrate mixed stones, which can form in the presence of hypercalciuria and a urinary pH above 6, are the most common type of renal stones in patients with dRTA. An estimated 35% of adult patients with calcium phosphate stones and urinary pH above 6.0 suffer from dRTA [4]. Distal RTA diagnosis requires an acidification test, an acid overload test or the furosemide test [5,6]. The best treatment for dRTA in adults is not well established; however, oral bicarbonate or oral potassium citrate, which alkalinize the internal environment, are commonly used. These treatments decrease metabolic acidosis, high bone resorption, and hypercalciuria.

Phytate, also known as inositol hexaphosphate (InsP6), is used by many plants for the storage of phosphorus and is most abundant in whole grains, legumes, and nuts, especially walnuts [7]. It has been shown that phytate ingestion decreases the risk of formation of some types of kidney stones, decreases the risk of development of cardiovascular calcifications [8] and also protects against osteoporosis [9,10,11]. The mechanisms by which these effects are generated are, however, complex. The phytate molecule is absorbed to a small extent by the intestine (as bisphosphonates) by paracellular transport. However, in the human intestine, although there are no own phytases, there may be phytases from food, which will degrade part of the phytate, resulting in lower inositol phosphates (InsPs) that can also be absorbed. On the other hand, both the absorbed InsP6 and the lower InsPs can be metabolized in the liver and in blood by other alkaline phosphatases [8]. As a result of all these processes, there is a diversity of InsPs molecules in the organism, some of them with the capacity to act as inhibitors of the crystallization of calcium salts (like bisphosphonates) and with possible synergistic effects among them. These molecules may also participate in other cellular processes, for example inhibiting osteoclastogenesis and mineralization of osteoblasts [10,11]. On the other hand, it has been shown that the oral intake of InsP6 significantly increases the excretion of InsPs [8].

When supplementing with phytate for the treatment of osteoporosis, the phytate salt used should be considered. Thus, it is very important to take into account that phytate is naturally present in plant seeds, which is its main source of supply in human food, in the form of calcium magnesium salt, which already involves a contribution of calcium to the diet, so it is unlikely to generate a deficiency in this element. In fact, the consumption of nuts and legumes is recommended for people with osteoporosis, and these substances contain significant amounts of phytin (calcium magnesium salt of phytate). However, if phytate is administered in the form of sodium salt, which is not the form usually found in nature, its effects may be clearly different, since the supply of significant amounts of sodium phytate, on the one hand, may influence the formation of insoluble calcium phytate in the intestine, with the consequent loss of calcium, and the excess of sodium, in addition to other undesirable effects, may increase the loss of calcium through urine [12]. In fact, in the first studies on the effects of phytate with experimental animals, carried out by Mellamby et al. in the 1940s, in which large doses of sodium phytate were administered to dogs, it was found that these animals developed significant rickets [13].

In the present paper, we examined patients with incomplete dRTA who were unable to tolerate treatment consisting of potassium citrate/bicarbonate and/or thiazide. Our main objective was to evaluate the effect of calcium magnesium phytate intake on calciuria in patients with incomplete dRTA.

## 2. Materials and Methods

### 2.1. Subjects and Study Design

This single-arm, interventional, pilot study prospectively enrolled patients from the outpatient clinic of the Urology Department of the University Hospital Son Espases. The inclusion criteria were: (i) positive diagnosis of dRTA based on a furosemide test; and (ii) discontinuation of treatment with potassium citrate, bicarbonate, or thiazide due to adverse effects. The exclusion criteria were pregnancy or positive urine culture. All included patients received endoscopic or extracorporeal lithotripsy treatment for urolithiasis in the Urology Department.

### 2.2. Dietary Intervention

Patients received a calcium magnesium phytate dietary supplement that consisted of 1 capsule (380 mg) of calcium magnesium InsP6 (Broken, SALVAT Laboratories S.A., Barcelona, Spain) every 12 h, which was taken on an empty stomach.

### 2.3. Variable Outcomes

The following variables were collected: age, sex, type of lithiasis, bilaterality of lithiasis, 24 h calciuria at baseline, and 24 h calciuria after 6 or more months of calcium magnesium phytate treatment. For one week prior to the furosemide test, all patients suspended all medications that could alter urinary pH. The dRTA test was performed by administering 40 mg of oral furosemide and measurement of urinary pH using a pH meter (Crison pH 25) for each urination episode during the following 3 h. A pH above 6 in all measurements was considered a positive result of dRTA diagnosis.

Urinary calculi were separated into the following four groups: hydroxyapatite calculi (HAP), hydroxyapatite and calcium oxalate dihydrate mixed calculi (HAP + COD), calcium oxalate dihydrate calculi (COD), or calcium oxalate dihydrate and calcium oxalate monohydrate mixed calculi (COD + COM). The study of kidney stones was performed with a MOTIC SMZ-161 stereoscopic microscope (MoticEurope, Barcelona, Spain), a Hitachi TM4000 Plus II desktop scanning electron microscope (Hitachi, Tokyo, Japan) coupled with Quantax 75 EDS microanalyzer (Bruker, Berlin, Germany), and a Bruker Hyperion IR spectrometer (Bruker, Berlin, Germany), with which the identification of major and minor components, the characteristics of internal and external structures, and information about areas with biological structures, such as renal tubules, can be obtained.

The bilaterality of the lithiasis was determined by review of the imaging results (abdominal X-ray, renal ultrasound, and abdomino–pelvic CT scan) from each patient’s medical history. Recurrence, defined as one or more episodes of renal colic after treatment or resolution of the first one, was established by a review of medical history, radiological tests, and clinical interviews.

The 24 h urine was collected in a sterile container with thymol as preservative agent. After collection, the total volume of the sample was recorded and an aliquot was used for calcium determination by atomic emission spectroscopy. The calciuria was measured at the beginning of the study (baseline) when patients were not receiving any pharmacological treatment (potassium citrate, bicarbonate, thiazides or phytate), and 6 months after initiation of treatment with dietary calcium magnesium phytate supplement.

### 2.4. Statistical Analysis

Values are expressed as mean ± standard deviation (SD) or frequency (percentage). The normality of data distributions was analyzed using normality plots and the Shapiro–Wilk test.

Intragroup comparisons (24 h calciuria before phytate vs. after phytate) were performed using a paired-samples *t*-test. Pearson’s correlation analysis was used to analyze the relationships between variables. Absolute change in calciuria was calculated as (calciuria at baseline)—(calciuria after calcium magnesium phytate treatment); the percentage decrease in calciuria was calculated as [(calciuria at baseline—calciuria after calcium magnesium InsP6 treatment)/calciuria at baseline] × 100%. All data were analyzed using IBM SPSS Statistics version 21. A two tailed *p*-value less than 0.05 was considered significant.

### 2.5. Ethical Considerations

The study was conducted according to the guidelines of the Declaration of Helsinki, and approved by the Research Committee of University Hospital Son Espases (protocol code CI-498-21, approved on 2021) and the Research Ethics Committee of the Balearic Islands (protocol code IB-4979/22, approved on 2022). All patients provided written informed consent before participation.

## 3. Results

**Figure 1:**
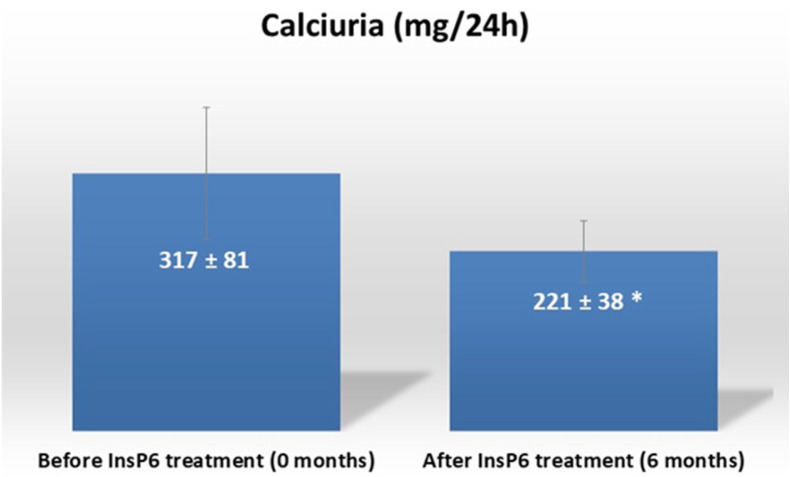
Calciuria before and after treatment with calcium magnesium phytate (InsP6). Values are expressed as mean ± SD. Values were compared using a paired-samples *t*-test. *: *p* < 0.05 vs. before treatment.

The study included 16 patients (8 men and 8 women) who had incomplete dRTA and were unable to tolerate conventional treatment. The main cause for discontinuing treatment with citrate was the appearance of side effects related to gastrointestinal problems. The formula used by these patients was a sustained-release formulation using a wax matrix, which mitigates these intestinal discomforts but does not eliminate them completely, probably due to the high doses used for the treatment of renal tubular acidosis (30–60 mEq/day). The mean (±SD) age was 47 years (±14) and 15 patients (93.8%) had bilateral urolithiasis (Table 1). Seven patients (43.8%) presented with COD + HAP (43.8%), six patients (37.5%) with HAP, two patients (12.5%) with COD + COM, and one patient (6.3%) with COD. The 24 h calciuria decreased from 317 (±81) mg/24 h at baseline to 221 (±38) mg/24 h at 6 months after oral calcium magnesium phytate treatment (*p* < 0.005, Figure 1). None of the patients experienced adverse effects.

**Figure 2:**
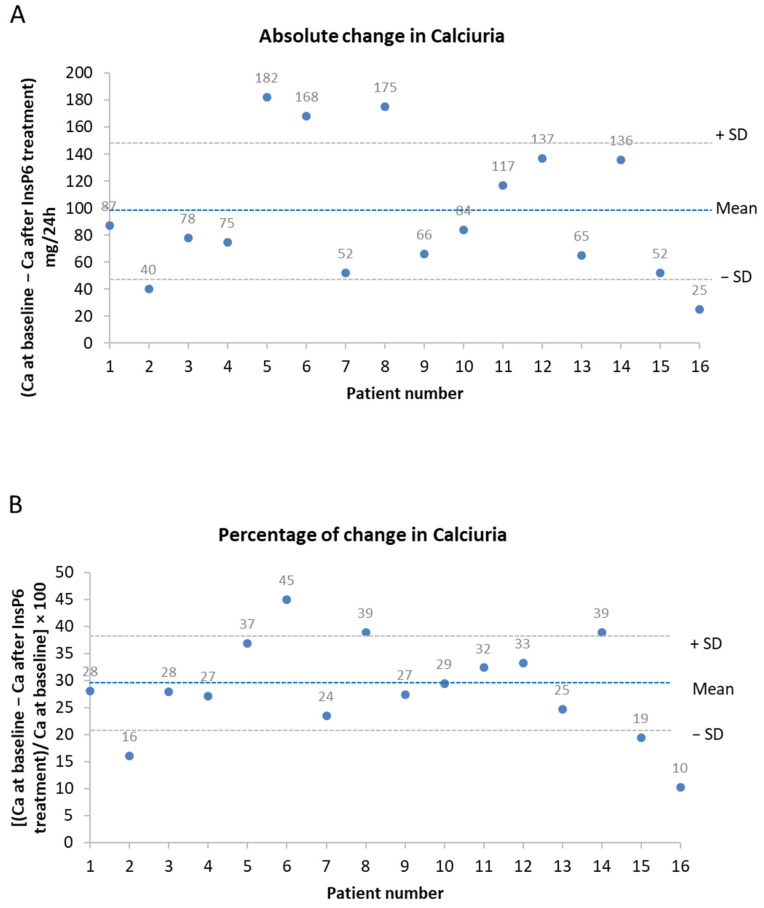
Absolute change (**A**) and percentage decrease (**B**) in calciuria after treatment with calcium magnesium phytate (InsP6) for all patients. Values are expressed as absolute change (Ca 0 months-Ca at 6 months) or percentage of change [(Ca 0 months − Ca at 6 months)/Ca at 0 months] × 100.

**Figure 3:**
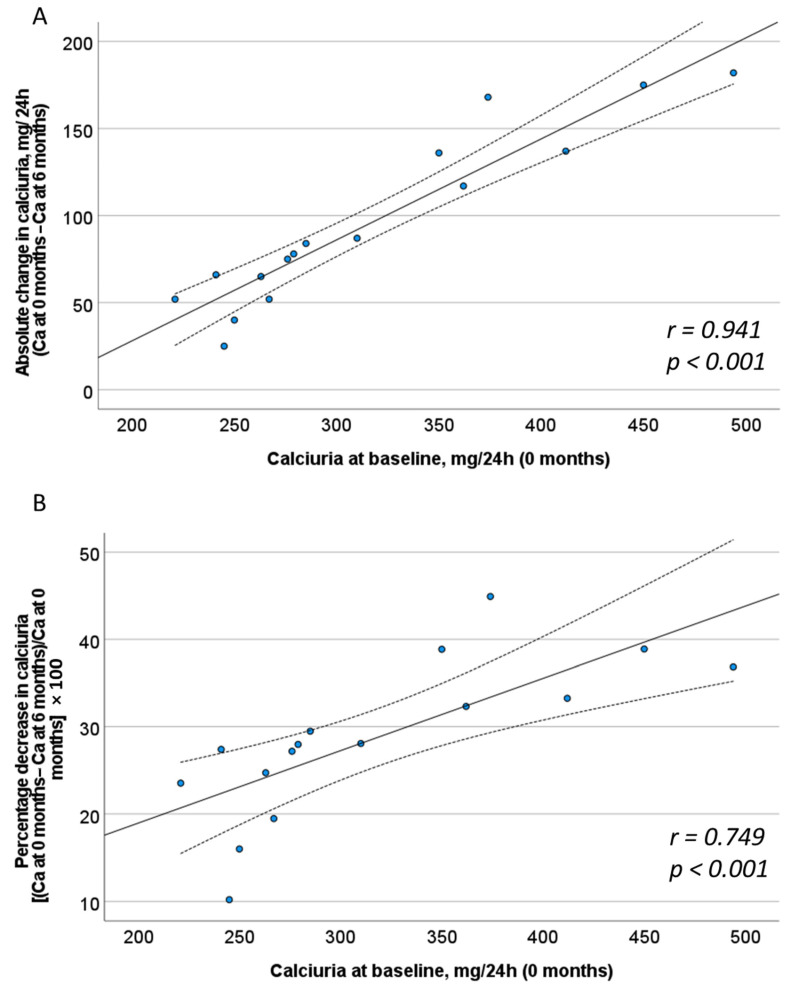
Correlation between absolute change (**A**) and percentage decrease (**B**) in calciuria after treatment with calcium magnesium phytate (InsP6) with calciuria at baseline. The solid line is the line best fit. The dotted lines are its 95% confidence bands. Pearson correlation coefficient and *p*-value are indicated in the lower right corner.

We also analyzed the absolute change and the percentage decrease in calciuria after treatment in individual patients (Figure 2). Notably, all patients experienced a decrease in calciuria after 6 months of calcium magnesium phytate treatment. The mean (±SD) absolute decrease was 96 (±50) mg/24 h, and the range was 25 to 182 mg/24 h (Figure 3A). The mean percentage decrease in calciuria was 29% (±9%), and the range was 10% to 45% (Figure 3B).

We also determined the correlation of different baseline characteristics with the decrease in calciuria after treatment with calcium magnesium phytate (InsP6) when calculated as an absolute change (Figure 3A) and as a percentage change (Figure 3B). The results showed that the greatest decreases in calciuria were in patients who had higher baseline levels of calciuria. Thus, there were significant correlations of baseline calciuria with absolute decrease in calciuria (r = 0.941; *p* < 0.001) and with percentage decrease in calciuria (r = 0.749; *p* < 0.001). However, there were no significant correlations of age or gender with the decrease in calciuria.

## 4. Discussion

Incomplete dRTA is the predominant form of dRTA in adults, but this condition is not well studied and there is no well-established treatment regimen for adults with incomplete dRTA [14]. The treatments are generally potassium citrate, bicarbonate, and thiazide-type diuretics [14,15]. However, adults with incomplete dRTA present with highly variable clinical manifestations due to the complex etiology of the disease. This disease is caused by alterations in α-intercalated cells of the collecting tubules within the nephrons due to variable degrees of dysfunction in the proton pumps (H^+^/K^+^ ATPase and vacuolar H^+^ ATPase) that excrete H^+^ into the urine [16]. Although there is no definitive treatment regimen, varying doses of potassium citrate are often used to treat incomplete dRTA due to its ability to alkalinize the patient’s internal environment and chelate urinary calcium. This treatment reduces the tendency for metabolic acidosis and bone resorption, and also reduces the availability of urinary calcium to form renal calculi [17].

However, 30% to 40% of patients may discontinue potassium citrate treatment, usually due to adverse gastrointestinal effects and lack of motivation [18,19]. We analyzed 16 highly motivated patients who had incomplete dRTA and abandoned treatment with potassium citrate and thiazides due to adverse effects. Previous studies suggested the use of phytate for these patients, given its tolerability and antiresorptive effects on bone [10,20]. At baseline (in the absence of dietary advice or treatment with potassium citrate or thiazide), our patients had a mean urinary calcium excretion of 317 ± 81 mg/24 h. After 6 months of treatment with calcium magnesium phytate, the mean urinary calcium excretion was 221 ± 38 mg/24 h (*p* > 0.005). Due to the short duration of our study, we were unable to examine the possible recurrence of urolithiasis. High urinary pH and hypercalciuria are the main causes of urolithiasis in patients with incomplete dRTA [2]. Because alkaline urine (pH > 6) cannot be easily corrected in patients with dRTA, a decrease in calciuria is likely the most effective intervention for decreasing the recurrence of urolithiasis in these patients. We believe that phytate and its metabolites decreased calciuria in these patients due to its antiresorptive effects on bone. In addition, the presence of phytate and its metabolites in the urine can inhibit the crystallization of calcium phosphate and calcium oxalate [8]. All of this implies that calcium magnesium phytate, given alone or in combination with potassium citrate and/or thiazides, can be considered a safe and effective treatment for patients with incomplete dRTA.

In this study, a previously described classification of renal calculi was used because of its simplicity and because of the close association of the different lithiasis categories with urine chemistry. Most of our patients had mixed COD + HAP stones (43.8%) and HAP stones (37.5%). Calcium phosphate stones, such as HAP, are more likely to form in urine with pH > 6.2 when there is hypercalciuria. However, the formation of COD stones does not require a high pH, but it does depend on hypercalciuria. Previous studies reported that the most common renal stones in patients with dRTA are calcium phosphate (HAP and brushite) and COD [4,21], in agreement with our results. However, two of our patients presented with mixed COD + COM stones. COM stones usually form when there is a deficiency of crystallization inhibitors, and do not require an alkaline urine or hypercalciuria. Electron microscopy of the stones of these two patients demonstrated that the COM crystals derived from the transformation of COD. When COD stones remain in the urinary tract for a long time, they can partially transform into COM stones [22].

We also found that 15 of our patients (93.8%) had bilateral urolithiasis. This coincides with previous studies which reported a high prevalence of stone bilaterality in patients with incomplete dRTA [4,23]. Distal RTA disease is a disorder that affects both kidneys simultaneously, so bilateral involvement of urolithiasis is to be expected. Recurrence was found in 100% of the patients of our sample. Recurrence in patients with dRTA is very frequent [4]. The main causes are that dRTA treatments are not curative, so the disease continues to affect the body, and that dRTA is often an underdiagnosed disease for which the correct treatment is not prescribed. In our sample, all recurrences occurred before the diagnosis of dRTA. Due to the short follow-up period and although no patient has suffered a recurrence, we cannot draw any conclusions about it. Since bilateral urolithiasis and formation of COD stones are risk factors for recurrence in urolithiasis [24], it is important to study these patients in detail because they may suffer from dRTA, in which case, they may benefit from appropriate medical treatment.

The gold standard for the diagnosis of dRTA is the acid overload test. However, we used the furosemide test for several reasons. It is simple to perform, with no side effects, and no significant dropout rate [4,5]; in contrast, the acid overload test is more complex and has a high rate of side effects and dropouts [5]. The studies carried out with the furosemide test for the diagnosis of dRTA have been applied to patients with urolithiasis taking into account factors such as bilaterality, type of kidney stone or urinary biochemistry. With an appropriate patient selection (urinary pH > 6.0, COD, HAP, or brushite stones), the furosemide and acid overload tests have equivalent sensitivity and specificity to the acid overload test [6,25].

Calcium magnesium phytate consumption has other beneficial effects for patients with dRTA presenting calcium oxalate and/or calcium phosphate renal calculi. Thus, among the many factors affecting the crystallization process, a very important one is the presence of additives, compounds other than the crystallizing product that can affect its crystallization in some way. In some cases, additives in insignificant concentrations change the speed of nucleation and growth, the size and/or morphology of the crystals formed, as well as the physical properties of the generated product. In general, it is assumed that different additives can act through different mechanisms. Thus, some additives selectively adsorb on certain crystal nuclei or crystal surfaces and block the active growth sites (steps, fractures, etc.); they are called crystallization inhibitors. In heterogeneous nucleation, an interaction occurs between the additive and the foreign solid phase that serves as a heterogeneous nucleant for the formation of the germ of the new phase. The additive can decrease the concentration of molecules of the substance that is nucleating on the surface of the heterogeneous nucleant. This decreases the catalytic effect of the foreign solid phase on nucleation, and the rate of formation of the germs of the new phase on the surface of the heterogeneous nucleant also decreases. This is reflected by the decrease in the total rate of heterogeneous nucleation. In the case of phytate, it is necessary to take into account that when it is orally or transdermal administered, a significant amount of its dephosphorylation products (InsPs, from InsP5 to InsP2, considering all its isomers) [8] are generated, which due to their different structure and location of the phosphate groups, can act in different ways, and may present important effects on some of the indicated processes.

The different molecules of InsPs formed can generally adsorb on the fracture of the growth step, along it, or on the surface between steps. In all cases, impediments are generated in the growth process so that a decrease in the growth rate takes place. Obviously, this effect is greater the stronger the adsorption strength of the additive to the surface. The additive units weakly adsorbed on the surface of the crystal remain in this state only for a short time, until they are desorbed, so their effects are reduced. It also depends on the supersaturation, since the greater it is, the lower the capacity of the additives or the higher their concentration needed for their effect to manifest.

There are additives that combine in solution with some of the species involved in the crystallization process. These substances, therefore, decrease the supersaturation of the substance and, consequently, also decrease its crystallization rate, although in this case, this decrease is not due to the interaction with the surface of the product that is generated, but due to an apparent increase in solubility as a consequence of the “disappearance” of one of the components that crystallize. In the case of phytate and its metabolites, formed as a consequence of its dephosphorylation, they do not directly alter the solubility of calcium salts in biological fluids, since although they can form complexes with calcium, they are found in concentrations around three orders of magnitude lower than those of this cation. However, it is very important to consider that since phytate is able to act by preventing bone resorption [9,20], as also confirmed in this paper, the concentration of calcium will decrease both at the plasma and urinary levels, and therefore, the supersaturation of the corresponding calcium salts will also decrease. This may be one of the most important aspects explaining the preventive action of calcium magnesium phytate intake in calcium lithiasis. It should be noted that while the inhibitory effect of a substance on the formation and development of crystals is time dependent, being a kinetic effect, the action on the decrease in supersaturation, being a thermodynamic aspect, is maintained over time, so it is surely a more relevant and transcendent effect than the kinetic one.

To our knowledge, this is the first study to examine the use of calcium magnesium phytate as a treatment for incomplete dRTA. The benefit of calcium magnesium phytate for these patients would probably be greater when it is combined with potassium citrate or thiazides. A limitation of this study is the short follow-up time; a longer follow-up time would allow assessment of the possible recurrence of urolithiasis. Another limitation is the small number of study participants. A further limitation of the study is that more than 90% of included patients had bilateral nephrolithiasis and recurrent stones, probably due to the aggressiveness of their condition, and that the study did not include patients with urolithiasis who were managed medically only. Recruitment of patients was difficult because dRTA is underdiagnosed in adults and because many patients tolerate the standard potassium citrate and/or thiazide treatment [26], making them ineligible for this study.

## 5. Conclusions

In conclusion, calcium magnesium phytate consumption significantly decreases calciuria in adult patients with incomplete dRTA. Future studies should compare conventional antihypercalciuric therapies with phytate treatment, as well as analyze the complementary effect of phytate when used together with conventional antihypercalciuric therapy.

## Figures and Tables

**Table 1:** Baseline characteristics of patients (*n* = 16). Values are expressed as frequency (percentage) or mean (±SD).

Age (Years)	47 (±14)
Gender:	
- Male - Female	8 (50.0%) 8 (50.0%)
Laterality:	
- Unilateral - Bilateral	1 (6.2%) 15 (93.8%)
Recurrence:	
- No: - Yes:	0 (0%) 16 (100%)
Type of lithiasis:	
- HAP + COD - HAP - COD + COM - COD	7 (43.8%) 6 (37.5%) 2 (12.5%) 1 (6.2%)

## Data Availability

The data that support the findings of this study are available from the corresponding author [A.C.-B.] upon reasonable request.
